# Identification of malaria transmission and epidemic hotspots in the western Kenya highlands: its application to malaria epidemic prediction

**DOI:** 10.1186/1756-3305-4-81

**Published:** 2011-05-19

**Authors:** Christine L Wanjala, John Waitumbi, Guofa Zhou, Andrew K Githeko

**Affiliations:** 1 Climate and Human Health Research Unit, Centre for Vector Biology and Control Research, Kenya Medical Research Institute, P. O. Box 1578 Kisumu 40100, Kenya; 2 Egerton University, P.O. Box 536, Nakuru, Kenya; 3 Kenya Medical Research Institute/Walter Reed Project, P. O. Box 1578 Kisumu 40100, Kenya; 4 Program in Public Health, College of Health Sciences, University of California, Irvine, CA 92697, USA

## Abstract

**Background:**

Malaria in the western Kenya highlands is characterized by unstable and high transmission variability which results in epidemics during periods of suitable climatic conditions. The sensitivity of a site to malaria epidemics depends on the level of immunity of the human population. This study examined how terrain in the highlands affects exposure and sensitivity of a site to malaria.

**Methods:**

The study was conducted in five sites in the western Kenya highlands, two U-shaped valleys (Iguhu, Emutete), two V-shaped valleys (Marani, Fort-Ternan) and one plateau (Shikondi) for 16 months among 6-15 years old children. Exposure to malaria was tested using circum-sporozoite protein (CSP) and merozoite surface protein (MSP) immunochromatographic antibody tests; malaria infections were tested by microscopic examination of thick and thin smears, the children's homes were georeferenced using a global positioning system. Paired t-test was used to compare the mean prevalence rates of the sites, K-function was use to determine if the clustering of malaria infections was significant.

**Results and Discussion:**

The mean antibody prevalence was 22.6% in Iguhu, 24% in Emutete, 11.5% in Shikondi, 8.3% in Fort-Ternan and 9.3% in Marani. The mean malaria infection prevalence was 23.3% in Iguhu, 21.9% in Emutete, 4.7% in Shikondi, 2.9% in Fort-Ternan and 2.4% in Marani. There was a significant difference in the antibodies and malaria infection prevalence between the two valley systems, and between the two valley systems and the plateau (P < 0.05). There was no significant difference in the antibodies and malaria infection prevalence in the two U-shaped valleys (Iguhu and Emutete) and in the V-shaped valleys (Marani and Fort Ternan) (P > 0.05). There was 8.5- fold and a 2-fold greater parasite and antibody prevalence respectively, in the U-shaped compared to the V-shaped valleys. The plateau antibody and parasite prevalence was similar to that of the V-shaped valleys. There was clustering of malaria antibodies and infections around flat areas in the U-shaped valleys, the infections were randomly distributed in the V-shaped valleys and less clustered at the plateau.

**Conclusion:**

This study showed that the V-shaped ecosystems have very low malaria prevalence and few individuals with an immune response to two major malaria antigens and they can be considered as epidemic hotspots. These populations are at higher risk of severe forms of malaria during hyper-transmission seasons. The plateau ecosystem has a similar infection and immune response to the V-shaped ecosystems. The U-shaped ecosystems are transmission hotspots.

## Background

**Figure 1:**
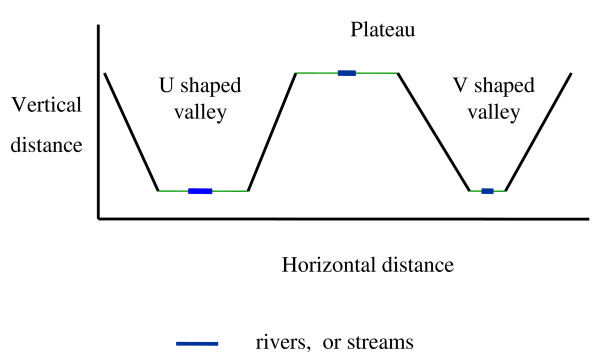
**Shape of the valley systems; this figure shows a sketch of the V-shaped valleys, U-shaped valleys and the plateau**.

Malaria epidemics have occurred in the highlands of western Kenya since the late 1980s, often resulting in high morbidity and mortality [1]. These epidemics have been associated with weather anomalies such as the El Niño phenomenon [2]. Apart from weather, other drivers of malaria transmission include topography [3] immunity [4] land use change [5] and drug resistance [1]. Entomological studies in different highland ecosystems indicated that transmission is heterogeneous. For example in ecosystems that are characterized by narrow valleys with fast flowing rivers the annual entomological inoculation rates (EIR) ranged from 0.4-1.1 infectious bites per person per year whereas, in ecosystems characterized by flat bottomed valleys with slow moving rivers, the annual EIR was 16.6 infectious bites per person per year [6]. While breeding of malaria vectors in the two ecosystems is confined to the valley bottoms, the broad shaped valleys have large flat surfaces where water can accumulate. In contrast, the narrow shaped valleys have relatively small flat surfaces providing stable breeding sites [7]. It has been shown that the productivity of a breeding habitat is a function of its stability [8]. The development of immunity to malaria is a function of the intensity and duration of exposure to infections. Measuring functional immunity to malaria remains a serious problem. However, proxies such as parasite density have been used to indicate suppression of parasitemia by the immune system. Major antigens linked to immune responses have been used as makers of the immune response. Studies in western Kenya indicate that areas of unstable transmission in the highlands, the prevalence of circumsprozoite protein (CSP) was 13% in adults over 40 years of age whereas in the stable transmission lowlands, approximately 65% of children were antibody positive [4]. Thus, the human population in the highland site has fewer people with immunity and this renders them vulnerable to severe forms of malaria during epidemics. The level of malaria transmission may also affect the production of gametocytes and the infectious reservoir of malaria. A large reservoir of infections would make available gametocytes to malaria vectors leading to stable and continuous transmission. The highlands were classified into three ecosystems, these being the flat bottomed valleys (U-shaped) the narrow bottomed valleys, (V) shaped and the plateau (Figure 1). We carried out a longitudinal cohort study with a primary focus on a spatial-temporal qualitative assessment of exposure to infections using immunological makers in the different ecosystems. Parasitological surveys were carried out to provide baseline data on the effects of ecosystem characteristics on malaria prevalence. Malaria epidemics in western Kenya highlands are driven by climate variability. However terrain characteristics can modify the level of malaria transmission and the rate of development of immunity. The risk of an epidemic is closely related to the level of immunity of the human population. While malaria epidemic prediction models [2] can identify the weather risks, the final outcome of the epidemic will be closely linked by the proportion of people who may not have had exposure to the disease and who have no immunity to malaria. This study was designed to identify how major terrain characteristics that control the breeding of malaria vectors in the western Kenya highlands can influence exposure to transmission and the development of an immune response. Identification of vulnerable populations in epidemic hotspots will make the epidemic prediction more spatially accurate.

## Methods and materials

### Study Sites

**Figure 2:**
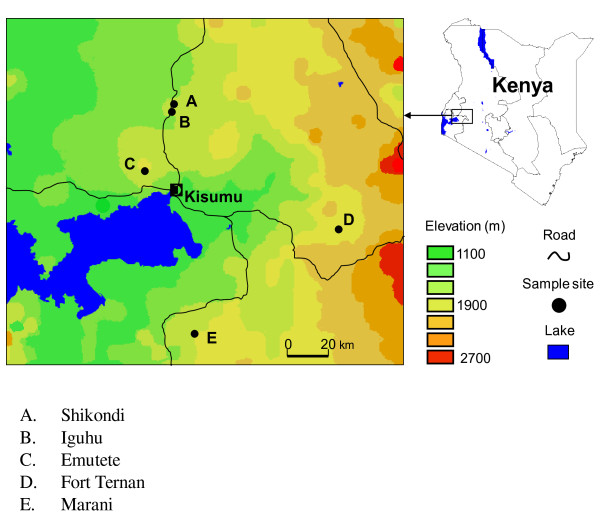
**The map of the five study sites; Iguhu, Emutete, Fort Ternan, Marani and Shikondi in Western Kenya**.

This study was conducted in five sites, two U-shaped valleys (Iguhu and Emutete), two V-shaped valleys (Marani and Fort Ternan) and one plateau (Shikondi). Iguhu (0°17'N, 34°74'E, and elevation 1,450-1,580 m above sea level) is located in Kakamega district, western Kenya. The slow flowing river Yala runs through the flood prone flat valley. Emutete village (34°64 E, 00°22 N, elevation 1,463 - 1,603 m above the sea level) is located in Emuhaya district in Western Kenya. The area is characterized by large flat bottomed valley with slow running streams. Marani (34°48 E, 00°02 N and elevation 1,520-1,700 m above the sea level) is located on the highland plateau adjacent to the Lake Victoria Basin and 17 km north of Kisii town. The fast flowing Marani river runs along the shallow gorge. Fort Ternan (00°12 S 35°21 E, elevation 1,500 m - 1,600 m above the sea level) is located in Kericho District. River Kipchorian bisects the hills and runs a long a deep gorge. Shikondi (0.192300 N, 34.763000 E, and elevation 1,533 m - 1,576 m above the sea level) is a plateau located 4 Km from Iguhu in Kakamega district, western Kenya (Figure 2)

### Sample size calculation

Because malaria prevalence in the study area was not well known, the sample size calculation was carried out assuming the prevalence was 50%. The sample size was calculated to achieve a 95% confidence and precision level of 5%.

The sample size required was 384 children from all the study sites, i.e. 77 children per site. Assuming a truancy of 10%, a total sample size of 425 children was obtained from a population of 600 children (120 from each site) who had given consent to participate in the study. The homes of the 600 children were geo-referenced, mapped, and tested for clustering. The human population density in all the study sites is very similar and so equal numbers of children were allocated to each site. Of the final sample size of 425 children, each site was allocated 85 children.

### Study Population

Cohorts of 170 children in the two U-shaped valleys, 170 children in the two V-shaped valleys and 85 children at the plateau aged 6-15 years were recruited for monthly *Plasmodium falciparum *surveys. The cohorts were followed monthly for 16 months (from May 2009- August 2010). Before the children were allowed to participate in the study, consent was obtained from their parents or guardians. Children aged between 6-15 years residing in the study sites and with no reported chronic illness, except malaria, were allowed to participate in the study. Children who were found having fever at the time of sampling were taken to the nearest government clinic for treatment.

### Immunoassays for exposure to malaria

The SCIMEDX (SCIMEDX Corporation, Deville, New Jersey, USA) rapid diagnostic antibody test kit contains a membrane strip, which is pre-coated with recombinant malaria *P. falciparum *capture antigens (MSP, CSP) on the test band *P. falciparum *region and with recombinant malaria *P.vivax *capture antigens (MSP, CSP) on the test band *P.vivax *region. The recombinant malaria *Plasmodium vivax (P.v)./Plasmodium falciparum (p.f). *antigen (MSP, CSP) -colloidal gold conjugate and serum sample moves along the membrane chromatographically to the test region *(P.f*, or *P.v.) *and forms a visible line as antibody-antigen gold particle complex forms with 91.3% sensitivity and 98.5% specificity. The control line is used for procedural control. Blood samples were collected by a standard finger prick and drawn into capillary tubes, then centrifuged at 1,800 revolutions per minutes for 5 minutes to obtain the serum. After the test kit had been brought to room temperature, a drop of serum was added in to the sample window and allowed to soak in, and then two drops of the diluent were added into the sample window. The positive results were read after 10 minutes and the negative results confirmed after 20 minutes as indicated by the kit manufacturer.

### Microscopy for the presence of the malaria parasites

Blood samples were collected by the standard finger-prick method and thick and thin smears prepared on labeled slides. The smears were allowed to air dry then fixed in methanol and stained in 4% Giemsa for 30 min. The stained smears were then examined using the magnification of × 1,000 (oil immersion) to identify and count the parasite species. Random checks were carried out on the slide counts by independent microscopists to ensure quality control. Parasite density was scored against 300 leukocytes in positive slides. Parasite densities were converted to number of parasites per microliter of blood, assuming a leukocyte count of 8,000 cells/μl [8].

### Spatial distribution maps

The homes of the participants were geo-referenced using a global positioning system (GPS) unit. A database was created with participants' names, ages, GPS location, and malaria parasite infection results. Participants' names were coded for confidentiality. ArcView 3.3 (Environmental Systems Research Institute, Redlands, CA, USA) was used to create spatial-distribution maps of infected and uninfected participants.

### Weather data

Mean monthly rainfall, maximum and minimum temperature for the study sites was obtained from the Kenya Department of Meteorology.

### Data analysis

Site specific prevalence rates were determined by expressing positive blood smears as a percentage of the total blood smears examined. Only positive slides were considered when the geometric mean parasite density was calculated for each site. The paired t-test was used to determine differences of the prevalence of antibody and malaria parasite infections among the sites. To determine spatial distribution patterns of malaria infections in the study area, ArcView 3.3 (Environmental Systems Research Institute, Redlands, CA, USA) was used to create Spatial-distribution maps of infected and uninfected participants for the 5 sites. *Plasmodium falciparum *infections were tested for clustering by household with the global spatial statistic, the K function, weighted by parasite density [9-11] by using Point Pattern Analysis software (San Diego State University, San Diego, CA, USA). The global weighted K function, *L*(*d*), examines the spatial dependence of malaria infection by household over a wide range of scales of pattern [12]. The observed *L*(*d*) function values were tested against the null hypothesis that the spatial distribution of all infected residents in the study area was random, by using 1,000 Monte Carlo iterations. The global spatial cluster analysis was conducted separately for the five sites.

### Ethical considerations

Scientific and ethical clearance was given by Kenya Medical Research Institute. Inclusion criteria were provision of informed consent, age >5 years at recruitment, and no reported chronic or acute illness, except malaria.

## Results

### Weather data from the meteorological stations near the study sites

Rainfall among the three districts, Kakamega, Kisii and Kericho were not statistically different. However Kisii received 435 mm more rainfall than Kakamega (Table 1). The mean annual maximum and minimum temperatures were significantly different among the sites with Kakamega being warmest and Kericho coolest (Table 1). The mean annual temperature between Kakamega and Kisii was not significantly different whereas it was significantly different between Kericho and Kisii and between Kakamega and Kericho.

**Table 1:** Summary of water data from meteorological stations near the study sites

	Values			P values		
**Sites**	**Kericho**	**Kisii**	**Kakamega**	**Kakamega vs Kisii**	**Kakamega vs Kericho**	**Kericho Vs Kisii**

Total annual rainfall (mm)	1,718.3	2,243.2	1,808.3	p > 0.05	p > 0.05	p > 0.05

Mean annual maximum temp °C	24.8	26.1	28.3	p < 0.05	p < 0.05	p < 0.05

Mean annual minimum temp °C	11.1	15.8	14.8	p < 0.05	p < 0.05	p < 0.05

Mean annual temperature °C	18.0	21.0	21.6	p > 0.05	p < 0.05	p < 0.05

Thus rainfall and mean annual temperatures were not significantly different between Kakamega and Kisii districts.

### Prevalence of *P. falciparum *malaria infections

**Figure 3:**
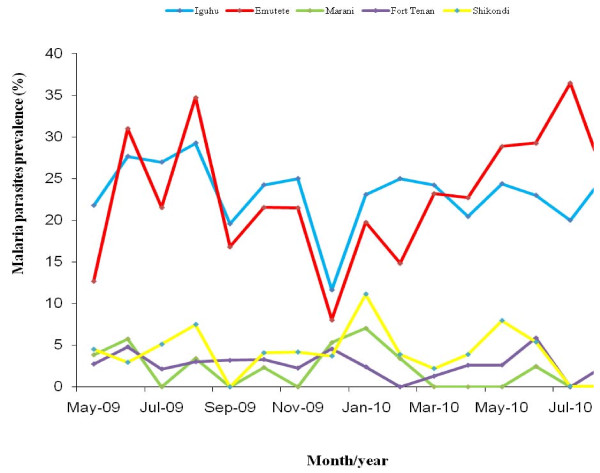
**The parasites prevalence was 8.5 fold higher in the in the U-shaped valleys (Iguhu and Emutete) than in the V- shaped valleys (Marani and Fort Ternan) and the plateau (Shikondi)**.

The mean *P. falciparum *prevalences during the 16 months study period were Iguhu, 23.3%, Emutete 21.9%, Marani 2.4% Fort Ternan 2.9% and Shikondi 4.7% (Figure 3). The prevalences in the two U-shaped valleys Iguhu and Emutete, were not significantly different (p > 0.05 t-test = 0.609) nor was the difference between the V-shaped valleys, Marani and Fort Ternan (p > 0.05, t-test = -0.672). The mean prevalences between the V-shaped valleys (Marani and Fort Ternan) and the plateau (Shikondi) were significantly different (p < 0.05, t-test = -2.47). No parasitemia were detected in Marani between March and May 2010. The difference in *P. falciparum *prevalence between the U and V-shaped valleys populations was highly significant (p << 0.05, t = 13.11) and so was the difference between the U-shaped valley populations and Shikondi the plateau (p << 0.05 t = 10.98). Seasonal variation in parasite prevalence was observed in populations living in the U- shaped valleys and was responsive to rainfall. Above normal El Niño rains were experienced in December 2009 which is normally a dry period (Figure 3).

### Mean parasite density in the five study sites

**Figure 4:**
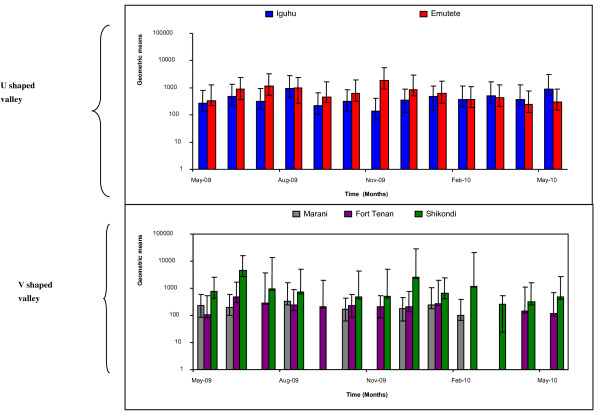
**The parasite density in the five sites, the geometric mean was the highest at the plateau (Shikondi) and showed high variation in the parasite density basing on the magnitude of the error bars**.

There was a significant difference in the geometric means of *P. falciparum *parasite density in the two U-shaped valleys; p < 0.05 t = -1.882 (Figure 4). There was no significant difference in the geometric means of *P. falciparum *parasite density in the two V-shaped valleys (Marani and Fort Ternan); p > 0.5 t = -1.678 (Figure 4). The mean parasite density and variance were highest at the plateau site of Shikondi and this was significantly different from the densities observed in the V-shaped valleys; p < 0.05 t = -2.584 (Figure 4).

### *P. falciparum *gametocyte prevalence in the five sites

In Marani, no gametocytes were detected throughout the study period, while in Fort Ternan the mean prevalence was 0.8%, Shikondi 1%, Iguhu 2.8% and Emutete 3.3%.

The gametocyte prevalence between Iguhu and Emutete (U-shaped valleys) was not significantly different (p > 0.05 t = -0.765), but was significantly different between the U-shaped valleys and V-shaped valley (Fort Ternan) (p < 0.05 t = 4.064) and between the U-shaped valleys and the plateau, Shikondi (p < 0.05 t = 5.058). However the prevalence between the V-shaped (Fort Ternan) and the plateau, Shikondi was not significantly different (p > 0.05, t = 0.463) (Table 2).

**Table 2:** The prevalence of gametocytes in the five sites

Month	Site				
	**Iguhu**	**Emutete**	**Fort Ternan**	**Marani**	**Shikondi**

**Prevalence %**					

**May-09**	1.0	4.2	0.9	0.0	0.0

**Jun-09**	6.5	2.1	1.0	0.0	1.0

**Jul-09**	2.1	1.7	0.0	0.0	1.0

**Aug-09**	3.4	5.2	0.0	0.0	2.7

**Sep-09**	4.7	3.0	0.0	0.0	0.0

**Oct-09**	2.0	2.9	0.0	0.0	1.0

**Nov-09**	1.3	1.3	1.0	0.0	0.0

**Dec-09**	0.0	0.0	1.2	0.0	1.3

**Jan-10**	1.1	2.2	0.0	0.0	0.0

**Feb-10**	3.3	2.0	1.0	0.0	0.0

**Mar-10**	4.2	1.0	2.2	0.0	0.0

**Apr-10**	3.2	5.7	0.0	0.0	0.0

**May-10**	2.4	5.6	4.6	0.0	0.0

**Jun-10**	1.2	7.3	0.0	0.0	2.2

**Jul-10**	3.5	5.4	0.0	0.0	2.2

**Aug-10**	4.6	3.3	0.0	0.0	3.1

**Mean**	**2.8**	**3.3**	**0.8**	**0**	**1**

### Prevalence of CSP-MSP antibodies in the five study sites

**Figure 5:**
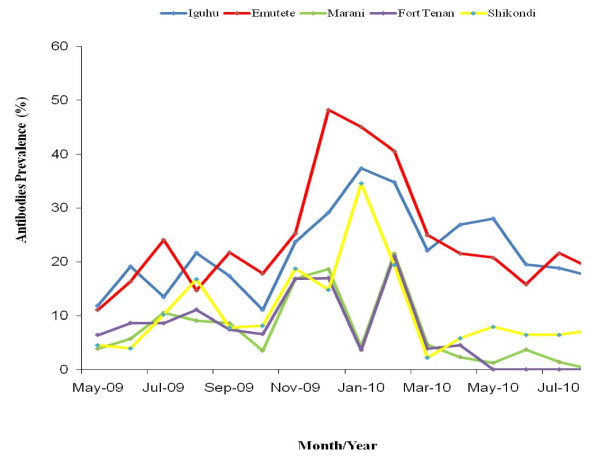
**The prevalence of CSP/MSP antibodies in the five studies, the antibodies prevalence was 2.6 fold higher in the U-shaped valleys (Iguhu and Emutete) than in the V-shaped valleys and the plateau**.

**Figure 6:**
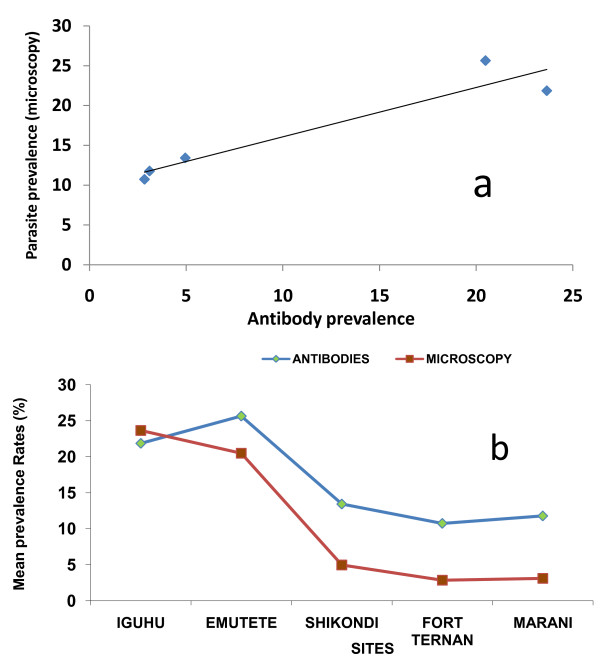
**(a) Positive correlation between antibody and parasite prevalence, (b) shows that the site-specific mean antibody prevalence increased with the site-specific mean parasite prevalence in all the study sites during the entire study period**.

The test kit for antibodies returned a positive result whether the individual responded to one or both antibodies. The mean prevalence of the antibodies in Iguhu was 22.6%, Emutete 24%, Marani 9.3%, Fort Ternan 8.3% and Shikondi 11.5%. There was a significant difference between the antibody prevalence in U-shaped valleys and the V-shaped valleys (p < 0.05, t = -6.226), and between the plateau and U-shaped valleys (p < 0.05, t = -6.182). There was no significant difference in antibody prevalence in the two U-shaped valleys (Iguhu and Emutete) (p > 0.05 t = -0.346), in the two V-shaped valleys (Fort Ternan and Marani) (p > 0.05 t = 0.352) and between the plateau (Shikondi) and the V-shaped valleys (p > 0.05, t = -0.889). An increase in antibody prevalence levels was observed after the rains in September 2009 and these declined rapidly in March 2010 (Figure 5). There was a positive correlation between site-specific mean antibody prevalence rate and mean site-specific malaria parasites prevalence rates, (Adjusted R^2 ^= 0.994; p = 0.00191) (Figure 6a) at the population level but not at the individual level. The mean site-specific prevalence of antibodies increased with the increase in the mean site-specific prevalence of malaria infections in all the study sites during the entire study period (Figure 6b).

### Spatial distribution of malaria cases

**Figure 7:**
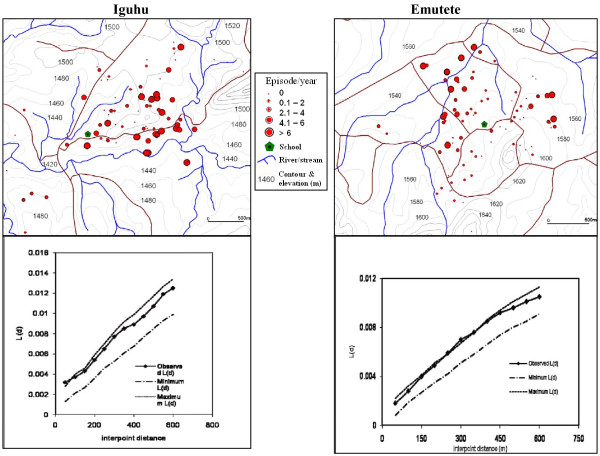
**The spatial distribution of malaria infections and K- function analysis in the "U" shaped valleys, malaria infections clustered around flat areas in the U-shaped valleys**. K-function spatial analysis shows that the clustering was significant.

**Figure 8:**
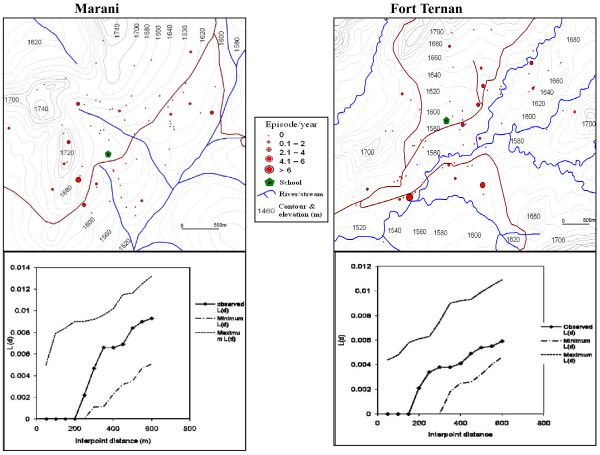
**Spatial distribution of malaria infections and K-function analysis in the "V" shaped valleys, but the infections are randomly distributed at the V-shaped valleys**.

**Figure 9:**
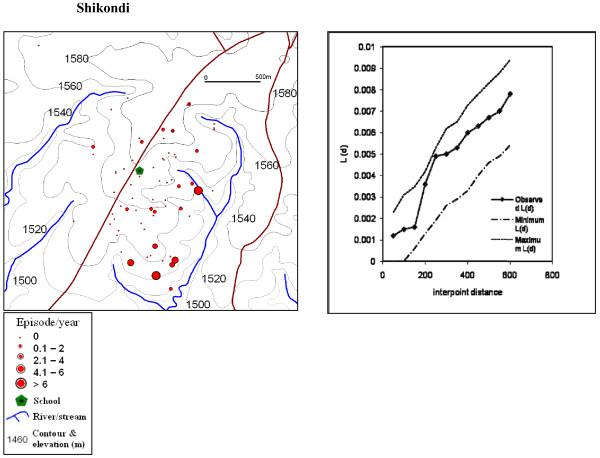
**Spatial distribution of malaria cases and cluster analysis at the plateau, there was less clustering at the flat areas of the plateau but it was not significant as shown in the K-function analysis**.

The global weighted K function, *L *(*d*), was used to examine the spatial distribution of malaria infections by household over an interpoint distance of 100-1,400 m for all the sites. Figure 7 shows measures of the observed *L *(*d*) and the 95% CI plotted for various values of interpoint distance for the surveys in Iguhu and Emutete respectively. The spatial distribution of infections was considered evenly dispersed if the observed K function values were below the lower limit of the 95% CI, clustered if above the upper limit, or random if within the 95% CI. The weighted K function indicated that the malaria infection distribution pattern was significantly different than expected under complete spatial randomness in the U-shaped valley but was random in the V-shaped valleys and the plateau (Figures 8 and 9). Spatial clustering occurred at flat areas in the U-shaped valleys (Figure 7); however it was not significantly clustered at the plateau (Figure 9) and was random in the V-shaped valley (Figure 8).

The majority of children with parasite positive blood smears in the U-shaped valleys were found infected on 4-6 occasions during 12 months while those in the V-shaped valleys and the plateau had 1-2 infections per year. Thus the children in the U-shaped valleys were infected for a longer period. (Figures 7 and 8)

## Discussion

Temporal variation of *P. falciparum *malaria prevalence in the highlands of western Kenya is a function of meteorological variables (rainfall, temperature and humidity) while the spatial variation is a function of the terrain characteristics. Furthermore these two drivers of malaria transmission have a direct impact on the development of an immune response to malaria infections. In this study we examine how the terrain characteristics are associated with prevalence levels of asexual and sexual stages of *P. falciparum *malaria spatial distribution of infections and the immune response. Knowledge gained has contributed towards differentiation of malaria epidemic and transmission hotspots which will further lead to a better understanding of the evolution of malaria epidemics.

Rainfall was similar among all the sites and the mean annual temperatures were not significantly different between Kakamega (Iguhu) and Kisii (Marani) which have U and V-shaped valleys respectively. Despite having similar weather, the two sites had very different *P. falciparum *malaria parasite prevalences, immune response and spatial distribution of malaria infections. The U-shaped valleys system had 8.5-fold more malaria infections and 2.6-fold greater prevalence of antibodies compared to the V- shaped valleys systems. This observation has implications in terms of predicting the outcome of weather-driven malaria epidemics and their prevention and control. In the highlands, most severe malaria cases during an epidemic come from human populations that have not been regularly exposed to malaria infections [13]. Physically the highlands of western Kenya are not a homogeneous ecosystem and have been shown to have a heterogeneous eco-epidemiology [3]. Thus while the climate may be similar in many places malaria infections also respond to terrain characteristics such as the shapes of the valleys which determine the availability and stability of vector breeding habitats and subsequently the level of malaria transmission and immunity. The outcome of a malaria epidemic is a direct function of the level of transmission and immunity.

The early malaria epidemic prediction model developed by Githeko and Ndegwa [2] detects temperature and rainfall anomalies that are supportive of increased vector populations and rapid sporogonic parasite development. It has been known that certain areas in the highlands are more prone to epidemics than others, but causes of this phenomenon were not clearly understood. In the western Kenya highlands the former Kisii, Kericho and Nandi districts have had a long history of severe malaria epidemics. We recognize that these districts have V-shaped valleys. In Kakamega district unpublished data (Ouna & Githeko unpublished) indicated a sharp increase in severe malaria cases at the plateau village of Shikondi during the 1997/8 El Niño driven epidemics. Malaria prevalence in this village is low (4.7%) and mean proportion of children with antibodies in this study was 11.5%, leaving a large proportion of the population with no detectable immune response.

During late 2009, El Niño type of rains started in September and continued to December. An increase in the proportion of children with antibodies increased by 24.2% in the U-shaped valleys system and this was associated with a 13.4% decrease in malaria prevalence in December. In the V-shaped valleys system an increase of 20.8% in the prevalence of antibodies was associated with only 1.7% decrease in malaria prevalence in November. These observations suggest that the immune response in the U-shaped valley systems may be more robust than that of the V-shaped valley systems. Besides a higher prevalence in malaria infections in the U-shaped valleys system, children were infected 3-fold more frequently than in the V-shaped valley system which may contribute to a better development of an immune response to malaria. Our results also showed a high positive correlation between site-specific mean antibodies and site specific mean parasite prevalence rates; this indicates that the site-specific malaria infection prevalence is predictive of the site-specific prevalence of CSP and MSP antibodies. While this relationship was strong at the population level, the relationship at the individual level was much less obvious and could have been affected by the sensitivity of kit, antigenic polymorphism and delayed immune response. Other studies have shown a log-linear relationship between exposure and antibodies prevalence predicted by mathematical models assuming exposure-dependent immunity [14]. The log-linear relationship between transmission intensity and prevalence of both infections and enlarged spleens thus support the existence of exposure-dependent acquired immunity [15].

The high gametocytes prevalence rates in the U-shaped valleys compared with the V- shaped valleys indicate that the reservoir of malaria infections in the U-shaped valleys is higher than in the V-shaped valleys and the plateau. While the population living in the U-shaped valley maintains a large reservoir of infectious gametocytes, the people living in the V-shaped valley comprise a high proportion of susceptible individuals. Under permissive climatic conditions the infectious vector population could increase, leading to higher rates of malaria prevalence. Earlier studies indicated that the mean gametocyte prevalence in Kericho district was 1.8% [16], 2.8% in Kakamega district [17]. In contrast gametocyte prevalence of 39.1% was reported in the holoendemic Kisumu District during the rainy season [18] and a rate of 6.6% in the mesoendemic Suba district, Western Kenya [19]. In our study the mean gametocyte density was significantly different between the V-shaped, the U-shaped valleys, and the plateau. The low prevalence of gametocytes in the V-shaped valleys with no gametocytes observed in Kisii indicates that there is a weak transmission system in the V-shaped valley.

Spatial analysis of the malaria antibodies and infections indicated that there was a significant positive clustering of malaria infections in the flat bottomed U-shaped valleys resulting from the high availability and stability of vector breeding habitats. In contrast malaria infections were randomly distributed in the V-shaped valleys, and this can be explained by the fact that these valley systems are characterized by fast running rivers at the valley bottoms, and steep slopes that provide good drainage in the area and so there are few vector breeding habitats in these ecosystems. We also observed less clustering in the plateau though it was not statistically significant. Earlier studies in the same area indicated that malaria was mesoendemic at the plateau at 26.7% prevalence [12]. Our results are consistent with studies at Iguhu which indicated that Anopheline larval habitats were generally clustered near the streams and rivers [20]. Similar studies have shown that the risk of malaria is strongly associated with distance from breeding sites [17]. Lower altitude within a highland area has been described in several studies as a risk factor for malaria [21,22]. Vector densities have been shown to cluster in low-lying flat areas [5], and reclaimed swamps [20,23].

Ongoing studies in the same areas indicate that the V-shaped valley ecosystems require anomalously high temperatures and rainfall over an extended period for epidemics to occur. These epidemics are defined by the numbers of people infected, severity of the disease and mortality. In Kericho district Western Kenya out of 254 malaria deaths 31% were due to cerebral malaria, 37% severe anemia and 32% due to malaria with gastroenteritis or pneumonia [24]. It is important to know which populations in the highlands are at high risk of severe disease during epidemics. Such areas require early and effective interventions to reduce high morbidity and mortality. Severe forms of malaria require hospitalization and treatment with quinine or blood transfusion and the demand on medical services can be tremendous.

As a strategy for epidemic prevention special attention should be focused on the V-shaped valley and plateau ecosystems as they constitute epidemic hotspots. It is likely that malaria could be eliminated in the current epidemic hotspots however the current transmission hotspots may convert to epidemic hotspots as the current malaria control efforts reduce transmission.

## Conclusion

This study has provided evidence that the V-shaped ecosystems have very low malaria prevalence and few individuals with an immune response to two major malaria antigens. This ecosystem can be regarded as an epidemic hotspot. These populations are a higher risk of severe forms of malaria during periods that support hyper-transmission of the parasites. When predicting malaria epidemics this factor should be taken into consideration. Plateaus with water bodies may also behave like the V-shaped valleys. It is critical that ecosystems sensitivities are taken into account in predicting and controlling malaria.

## Competing interests

The authors declare that they have no competing interests.

## Authors' contributions

CLW participated in the study design, supervision of the field work, data analysis and drafted the manuscript. AKG designed the study, extracted the rainfall and temperature data from the metrological stations contributed to writing the manuscript, GZ did the mapping of the study sites and spatial analysis, JW participated in the designing of the study and contributed to writing of the manuscript. All authors have read and approved the final manuscript.

## References

[B1] Hay SI Simba M Busolo M Noor AM Guyatt HL Ochola SA Snow RW Defining and detecting malaria epidemics in the highlands of Western Kenya Emerg Infect Dis 2002 8 555 562 12023909 10.3201/eid0806.010310 PMC2738480

[B2] Githeko AK Ndegwa W Predicting malaria epidemics using climate data in Kenyan highlands: a tool for decision makers Global Change and Human Health 2001 2 54 63 10.1023/A:1011943131643

[B3] Noland GS Hendel-Paterson B Min XM Moormann AM Vulule JM Narum DL Lanar DE Kazura JW John CC Low prevalence of antibodies to preerythrocytic but not blood-stage *Plasmodium falciparum *antigens in an area of unstable malaria transmission compared to prevalence in an area of stable malaria transmission Infect Immun 2008 76 5721 5728 10.1128/IAI.00591-08 18809666 PMC2583556

[B4] Afrane YA Lawson BW Githeko AK Yan G Effects of microclimatic changes caused by land use and land cover on duration of gonotrophic cycles of *Anopheles gambiae *(Diptera: Culicidae) in Western Kenya highlands J Med Entomol 2005 42 974 980 10.1603/0022-2585(2005)042[0974:EOMCCB]2.0.CO;2 16465737

[B5] Ndenga B Githeko A Omukunda E Munyekenye G Atieli H Wamai P Mbogo C Minakawa N Zhou G Yan G Population dynamics of malaria vectors in Western Kenya highlands J Med Entomol 2006 43 200 206 10.1603/0022-2585(2006)043[0200:PDOMVI]2.0.CO;2 16619599

[B6] Himeidan YE Zhou G Yakob L Afrane Y Munga S Atieli H El-Rayah el A Githeko AK Yan G Habitat stability and occurrences of malaria vector larvae in Western Kenya highlands Malar J 2009 8 234 10.1186/1475-2875-8-234 19845968 PMC2771030

[B7] Himeidan YE Malik EM Adam E Epidemiological and seasonal pattern of malaria in irrigated areas of Eastern Sudan Am J Infect Dis 2005 1 75 78

[B8] Slutsker L Taylor TE Wirima JJ Steketee RW In-hospital morbidity and mortality due to malaria-associated severe anaemia in two areas of Malawi with different patterns of malaria infection Trans R Soc Trop Med Hyg 1994 88 548 551 10.1016/0035-9203(94)90157-0 7992335

[B9] Getis A Franklin J Second-order neighborhood analysis of mapped point patterns Ecology 1987 68 473 477 10.2307/1938452

[B10] Haase P Spatial pattern analysis in ecology based on Ripley's K function: introduction and methods of edge correction J Veg Sci 1995 6 575 582 10.2307/3236356

[B11] Ripley B The second-order analysis of stationary point processes J Appl Probab 1976 13 255 266 10.2307/3212829

[B12] Githeko AK Ayisi JM Odada PK Atieli FK Ndenga BA Githure JI Yan G Topography and malaria transmission heterogeneity in western Kenya highlands: prospects for focal vector control Malar J 2006 5 107 10.1186/1475-2875-5-107 17096835 PMC1654174

[B13] Zhou G Minakawa N Githeko AK Yan G Association between climate variability and malaria epidemics in the East African highlands Proc Natl Acad Sci USA 2004 101 2375 2380 10.1073/pnas.0308714100 14983017 PMC356958

[B14] Gupta S Snow RW Donnelly CA Marsh K Newbold C Immunity to non-cerebral severe malaria is acquired after one or two infections Nat Med 1999 5 340 343 10.1038/6560 10086393

[B15] Bodker R Msangeni HA Kisinza W Lindsay SW Relationship between the intensity of exposure to malaria parasites and infection in the Usambara Mountains, Tanzania Am J Trop Med Hyg 2006 74 716 723 16687668

[B16] Ayisi JM Githeko AK Owaga ML Ekisa WS Anyona DB Obala AA Oloo AJ A survey of malaria endemicity in Kericho District, Kenya Proceedings of the 12th Annual Medical Scientific Conference of KEMRI/KERTI,. Nairobi 1991 242 246

[B17] Munyekenye OG Githeko AK Zhou G Mushinzimana E Minakawa N Yan G *Plasmodium falciparum *spatial analysis, Western Kenya highlands Emerg Infect Dis 2005 11 1571 1577 16318698 10.3201/eid1110.050106 PMC3366738

[B18] Githeko AK Brandling-Bennett AD Beier M Atieli F Owaga M Collins FH The reservoir of *Plasmodium falciparum *malaria in a holoendemic area of Western Kenya Trans R Soc Trop Med Hyg 1992 86 355 358 10.1016/0035-9203(92)90216-Y 1359683

[B19] Gouagna LC Okech BA Kabiru EW Killeen GF Obare P Ombonya S Bier JC Knols BG Githure JI Yan G Infectivity of *Plasmodium falciparum *gametocytes in patients attending rural health centres in Western Kenya East Afr Med J 2003 80 627 634 15018419 10.4314/eamj.v80i12.8779

[B20] Minakawa N Sonye G Mogi M Yan G Habitat characteristics of *Anopheles gambiae s.s. *larvae in a Kenyan highland Med Vet Entomol 2004 18 301 305 10.1111/j.0269-283X.2004.00503.x 15347399

[B21] Brooker S Clarke S Njagi JK Polack S Mugo B Estambale B Muchiri E Magnussen P Cox J Spatial clustering of malaria and associated risk factors during an epidemic in a highland area of Western Kenya Trop Med Int Health 2004 9 757 766 10.1111/j.1365-3156.2004.01272.x 15228485

[B22] Drakeley CJ Carneiro I Reyburn H Malima R Lusingu JP Cox J Theander TG Nkya WM Lemnge MM Riley EM Altitude-dependent and -independent variations in *Plasmodium falciparum *prevalence in northeastern Tanzania J Infect Dis 2005 191 1589 1598 10.1086/429669 15838785

[B23] Lindblade KA Walker ED Onapa AW Katungu J Wilson ML Land use change alters malaria transmission parameters by modifying temperature in a highland area of Uganda Trop Med Int Health 2000 5 263 274 10.1046/j.1365-3156.2000.00551.x 10810021

[B24] Malakooti MA Biomndo K Shanks GD Reemergence of epidemic malaria in the highlands of Western Kenya Emerg Infect Dis 1998 4 671 676 10.3201/eid0404.980422 9866748 PMC2640260

